# Manifold angles, the concept of self-similarity, and angle-enhanced bifurcation diagrams

**DOI:** 10.1038/srep18859

**Published:** 2016-01-06

**Authors:** Marcus W. Beims, Jason A. C. Gallas

**Affiliations:** 1 Departamento de Física, Universidade Federal do Paraná, 81531-990 Curitiba, Brazil; 2 Departamento de Física, Universidade Federal da Paraíba, 58051-970 João Pessoa, Brazil; 3 Instituto de Altos Estudos da Paraíba, Rua Infante Dom Henrique 100-1801, 58039-150 João Pessoa, Brazil; 4 Max Planck Institute for the Physics of Complex Systems, Nöthnitzer Str. 38, 01187 Dresden, Germany; 5 Institute for Multiscale Simulation, Friedrich-Alexander-Universität Erlangen-Nürnberg, 91052 Erlangen, Germany

## Abstract

Chaos and regularity are routinely discriminated by using Lyapunov exponents distilled from the norm of orthogonalized Lyapunov vectors, propagated during the temporal evolution of the dynamics. Such exponents are mean-field-like averages that, for each degree of freedom, squeeze the whole temporal evolution complexity into just a single number. However, Lyapunov vectors also contain a step-by-step record of what exactly happens with the angles between stable and unstable manifolds during the whole evolution, a big-data information permanently erased by repeated orthogonalizations. Here, we study changes of angles between invariant subspaces as observed during temporal evolution of Hénon’s system. Such angles are calculated numerically and analytically and used to characterize self-similarity of a chaotic attractor. In addition, we show how standard tools of dynamical systems may be angle-enhanced by dressing them with informations not difficult to extract. Such angle-enhanced tools reveal unexpected and practical facts that are described in detail. For instance, we present a video showing an angle-enhanced bifurcation diagram that exposes from several perspectives the complex geometrical features underlying the attractors. We believe such findings to be generic for extended classes of systems.

A research topic currently attracting growing interest is the exploration of certain vectors between the stable and unstable manifolds underlying the equations of motion of complex nonlinear systems like, e.g., lasers, chemical and biological oscillators, electronic circuits, and other systems. The main motivation is to obtain new insight about intricate aspects of complex systems (e.g. local diffusion of particles, sticky effects in conservative systems, etc) as well as pointwise properties of attractors. The key goal is to improve the ability of predicting stability in general, as well as to anticipate catastrophic events or regularities in the temporal evolution of phenomena of interest.

The primary tool used to characterize dynamical stability and to discriminate chaos from regularity is the so-called spectrum of Lyapunov exponents. Originally introduced by Lyapunov in 1892 in his doctoral thesis^1^,^2^,^3^,^4^, such spectrum is given by the limit eigenvalues of a suitable product of matrices. Technically, the computation of the Lyapunov spectrum is routinely done using the Gram-Schmidt orthogonalization procedure and involves determining the magnitude of expansion and contraction rates along the unstable and stable directions (defined^5^ by “singular vectors”) of the phase-space. The Gram-Schmidt procedure is efficient to extract Lyapunov exponents from the magnitudes of vectors but destroys all information concerning the relative angles between manifolds. Although the Lyapunov spectrum can be obtained from the limit eigenvalues of a suitable matrix, it is not possible to use the corresponding eigenvectors, because they are corrupted by the repeated Gram-Schmidt orthogonalization.

Presently, widespread attention is being attracted by powerful tools based on the so-called *Lyapunov vectors* (LVs)^5^,^6^,^7^,^8^,^9^,^10^,^11^,^12^, a class of vectors from which one can conveniently extract not only the familiar Lyapunov spectrum, but also much more informations. Specifically, several research groups are currently searching practical applications for the angles between LVs as vehicles to gain access to additional dynamical informations which cannot be obtained by other means. As pointed out by Wolfe and Samelson^5^, LVs are natural generalizations of normal modes for linear disturbances to aperiodic deterministic flows and provide insight concerning the physical mechanisms of aperiodic flow and the maintenance of chaos. LVs allow one to characterize the nature of the intersection between stable and unstable manifolds along trajectories. Such vectors can be obtained by iterating the equations of motion forward and backward along the same trajectory according to effective algorithms proposed only recently by Wolfe *et al.*^5^,^8^ and by Ginelli *et al.*^9^,^10^. In addition to these works, a nice overview of the theory and numerical algorithms is given by Kuptsov and Parlitz^11^ (see also Ng *et al.*^12^). Incidentally, when periodic orbits are considered, the Lyapunov exponents and vectors coincide with the (real part of) Floquet ones.

Lyapunov vectors have already been used to obtain insight for a number of practical situations. For instance, an early work of Pomeau, Pumir, and Pelce^13^ considered a partial differential equation, a model equation describing a system with infinite degrees of freedom, and investigated aspects of fully developed turbulence based on the distribution of Lyapunov numbers and the power spectrum of the associated LVs. More recently, Palatella *et al.*^7^ presented a proof-of-concept showing how the traffic state can be estimated using a data assimilation algorithm based on LVs and partial and noisy data obtained with current GPS technology by monitoring position and velocity of vehicles. They show that their algorithm is more efficient if the system is not chaotic and that an accurate reconstruction of the complete traffic state can be obtained at a very low computational cost by monitoring only a small percentage of vehicles. Norwood *et al.*^8^ reported a study of a toy ocean-atmosphere model coupling three Lorenz systems with different time scales, in order to test the effects of fast and slow modes of growth on the dynamical vectors. A fast ‘extratropical atmosphere’ is weakly coupled to a fast ‘tropical atmosphere’ which is, in turn, strongly coupled to a slow ‘ocean’ system, the latter coupling imitating the tropical El Niño–Southern Oscillation. Among other things, they find that LVs are able to successfully separate the fast extratropical atmosphere, but are unable to completely decouple the tropical atmosphere from the ocean, showing that LVs are mainly useful in the (slow) ocean system, but are affected by changes in the (fast) tropical system. Wolfe and Samelson^5^ studied LVs for the Lorenz model and the nonlinear Phillips model of the baroclinic instability, considered to be an extension of the familiar 1963 Lorenz model. Kobayashi and Saiki used LVs to characterize the parameter at which periodic windows corresponding to unstable periodic orbit finish^14^, and also analyzed the nonhyperbolicity of the Lorenz system^15^. Kuptsov and Kuptsova found that LVs are highly localized in scale-free networks of chaotic Hénon maps^16^. Our motivation is to develop tools to address certain problems that we faced recently in other complex dynamical settings^17^,^18^,^19^,^20^,^21^,^22^.

## Results

### Numerical determination of manifold angles

Previous works studied questions related mainly to hyperbolicity^23^ in several contexts^5^,^9^,^10^,^11^,^24^,^25^,^26^. Here, our aim is to use the LVs to explore the angles between invariant manifolds, and predictions derived from them, for the paradigmatic two-dimensional Hénon map


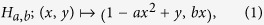


where *a* and *b* are real parameters and *x* and *y* are real variables^27^,^28^. This system was selected because, despite the simplicity of its equations of motion, it contains the basic mechanisms typically found in more complex systems, being therefore a convenient and realistic test-bed to prospect and learn about the capabilities of LVs and manifold angles. The map is particularly useful for our analysis because its low dimensionality avoids certain difficulties associated with calculating principal angles between higher-dimensional subspaces, something still under theoretical investigation^10^,^11^. More specifically, we use LVs to address questions related to the self-similarity of the Hénon attractor and related to the structural unfolding of bifurcations. We start by reviewing briefly standard knowledge obtained from the *geometrical* structure of the attractor. Then, we show what sort of new insight can be obtained from the angles between stable and unstable varieties and what such angles imply for the concept of self-similarity and the bifurcation diagram of the map. Furthermore, we determine exact analytical expressions for angles between stable-stable and stable-unstable manifolds for period–1 and period–2 orbital points as a function of the parameters (*a*, *b*). Results from our numerical procedure are in perfect agreement with the analytical ones for low-periodic orbits as long as the eigenvalues of the Jacobian matrix from Eq. (1) are real. For complex eigenvalues no stable or unstable directions exist, a feature nicely detected by the numerical procedure, as explained below.

**Figure 1:**
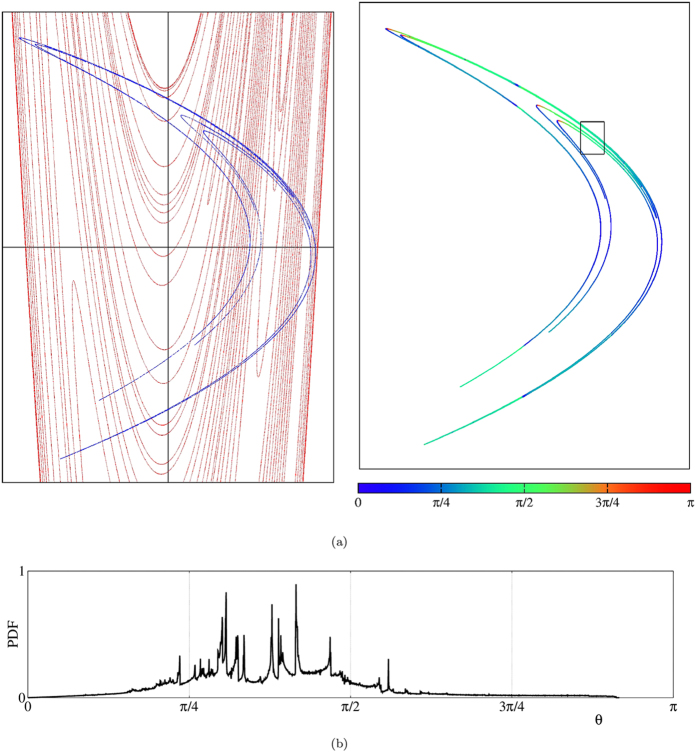
(**a**) Left panel: The blue points show the attractor for *a* = 1.4 and *b* = 0.3, plotted on top of part of the stable manifold (red segments) of the fixed point embedded in the attractor. (**a**) Right panel: The same attractor, now with colors displaying the angle between the covariant Lyapunov vectors (see text). (**b**) Distribution of the angles between stable and unstable manifolds.

Depending on parameter values and initial conditions, the Hénon map is well-known to display stable periodic attractors of arbitrary periods, or non-periodic attractors which, in some cases, for unphysically small values of *b*, were proved to be *chaotic*^29^. The *a* × *b* control space is characterized by specific paths displaying systematic organization and accumulations of stable oscillations^30^. The most famous attractor of the map is the chaotic *Hénon attractor* found for (*a*, *b*) = (1.4, 0.3) and illustrated in Fig. 1. For this parameter point, a key feature discovered by Hénon was an apparent *self-similarity* displayed by the attractor, consequence of its chaotic behavior. Here, we report strong numerical evidence suggesting that the original characterization of self-similarity, by looking solely at the layers of points composing the attractor, is misleading. The problem is that such layers (aligned along the direction of the unstable manifold) are not perfectly parallel among themselves. Therefore, what successive magnifications of the attractor show is, in fact, a sequential alternation of layers of points that approach and separate themselves. Here, we show that the Lyapunov vectors allow one to easily visualize such alternation and, in so doing, indicate a more accurate way to characterize self-similarity in the Hénon attractor. The continued angular alternation of the layers of points shows that, strictly, no self-similarity is possible for the Hénon attractor. In real-life, an *apparent* self-similarity emerges due to the impossibility of performing computations or measurements with infinite precision. In practice, the onset of such apparent self-similarity occurs very fast, due to the exponential convergence to the attractor. For brevity, from now on we refer simply to self-similarity, indistinctly.

The left panel of Fig. 1(a) shows the Hénon attractor (in blue) plotted together with a set of red lines depicting a portion of the dense stable manifold of the fixed point embedded in the attractor. Although no proof was ever found, the Hénon attractor is widely believed to lie along the unstable manifold. Accordingly, the angles between the blue and red lines should correspond to the angles between the two Lyapunov vectors, one along the unstable direction and the other one along the stable direction. From the left panel in Fig. 1(a) one may get a good qualitative understanding of the regions in phase-space where the manifolds are tangent and where they intersect transversally. Using the procedure described in the Supplementary Material, for each point of the attractor we computed the actual angle *θ*_*num*_ between the stable and unstable manifolds. Such angles are shown on the right panel of Fig. 1(a), where each point of the attractor is plotted using colors to represent the angle between the blue and red lines seen on the left panel of Fig. 1(a). As it is clear from the figure, the angles are distributed in the interval [0, *π*]. To emphasize how the manifolds approach the degenerate angles 0 and *π* we deliberately use two distinct colors at the ends of the colorbar. Comparing the upper panels of Fig. 1 one sees that the angles between the Lyapunov vectors reproduce compactly the angular trends between the manifolds. Finally, Fig. 1(b) illustrates the probability distribution function of the angle between the manifolds for each point of the Hénon attractor. The figure shows 80 × 10^3^ points for a bin width of 0.001. Such probability distribution function is a suitable tool to analyze the hyperbolicity of the system, as done e.g. in refs 9,24.

**Figure 2:**
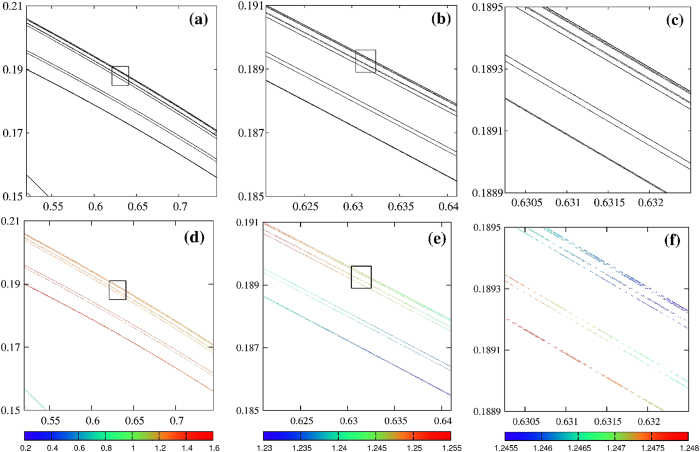
Successive magnifications displaying Hénon’s attractor in two distinct ways. Top row: Hénon’s original sequence, with magnifications of 10%. Hénon^27^ used these magnifications to argue for the self-similarity of the attractor. Bottom row: angle-enhanced views of the same graphs on the top panels evidencing the lack of self-similarity among angles between manifolds.

In his seminal work, Hénon reported an attractor consisting apparently of series of “curves” more or less parallel to each other^27^. The *longitudinal structure* of the attractor appears to be simple, each curve looking essentially like a one-dimensional manifold. In contrast, the *transversal structure* across the curves is entirely different and much more complex. To understand the nature of this structure, Hénon magnified the region contained in the box of the right panel in Fig. 1(a) together with two smaller subregions located inside this box, near the unstable fixed point embedded in the attractor. These three magnifications are adapted here in Fig. 2, top row. Figure 2(a) shows a magnification of the aforementioned box in Fig. 1 and contains what appears to be six thin layers of points. Next, a magnification of the rectangle seen in Fig. 2(a) is magnified in Fig. 2(b) and contains apparently three layers of points. This enlargement reveals the presence of six thin additional layers of points resembling the layers in Fig. 2(a). Further magnification of the box indicated in Fig. 2(b) leads to Fig. 2(c), which once more displays six thin layers of points, again structurally similar to the previous layers.

Hénon argued that such magnifications strongly suggest that the process of multiplication of “curves” will continue indefinitely, and that each apparent “curve” close to the fixed point is in fact made of an infinity of points arranged along quasi-parallel curves. Moreover, Fig. 2(b,c) indicate the existence of a hierarchical sequence of “levels”, their overall structure being practically identical at each level, apart from scale factors. In other words, the attractor seems to be self-similar because Fig. 2(a–c) look almost identical: each of them seems to display six thin layers despite the fact that they are obtained by magnifying successively regions that appear to contain no more than three layers of points. This cascading suggests the local structure of the attractor to be given by the product of a line with a Cantor set because transversal cuts through cross sections of chaotic attractors are known to frequently display such Cantor set structure^31^. However, a natural question is to ask if the bare visualization of the orbital points shown in Fig. 2(a–c) is enough to characterize self-similarity. Careful inspection of the attractor reveals that there are many regions where it is *not* self-similar. Manifold angles provide a simple way for quantifying to which extent attractors are self-similar or self-similarity breaks down.

Figure 2(d–f) depict the same orbital points in Fig. 2(a–c) but now including informations from the angles between the invariant manifolds. Using the color coding under each panel, these figures record the value of angle *θ*_*num*_ between the Lyapunov vectors for each point of the attractor. Analyzing the distribution of colors, we easily recognize from Fig. 2(d) that the three upper thin layers have angles *θ*_*num*_ *smaller* than the ones in the lower three thin layers seen in the same figure. In contrast, in Fig. 2(e) the situation is reversed, the upper three layers have *larger* angles than its lower layers. Next, in Fig. 2(f) we observe that the upper trio has *smaller* angles than the lower layers, repeating the situation seen in Fig. 2(d). Consequently, the sequential magnifications used by Hénon are clearly seen to break self-similarity when the manifold angles are taken into account. These observations cast doubts about the true nature of the self-similarity of the underlying attractor and, more generally, about the ability of such magnifications to detect self-similarity. Before addressing these points, we first elucidate the physical origin of the tiny differences that the Lyapunov vectors reveal to exist between the angles in the upper and lower layers on points in Fig. 2(d–f).

**Figure 3:**
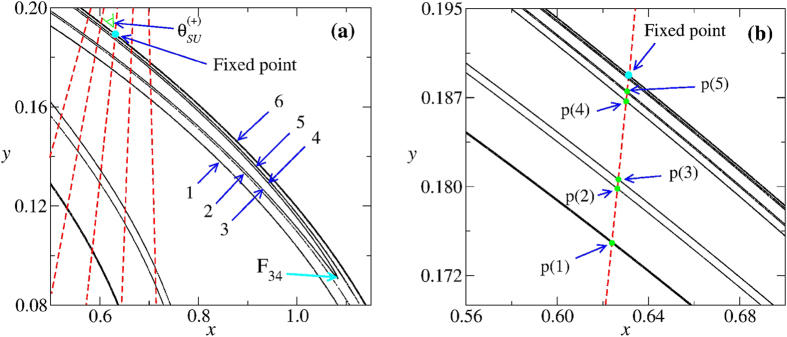
(**a**) Portion of the Hénon attractor illustrating that its several layers are not parallel to each other due to an infinite quantity of folds similar to *F*_34_. Black points show the Hénon attractor (unstable manifold). Red dashed lines represent schematically a few segments of the stable manifold of the fixed point indicated in the figure. The green mark on the left of the fixed point indicates one of the angles computed here, namely an angle between the stable manifold (red dashed lines) and the lines composing the attractor foliation. (**b**) Magnification near de fixed point indicating the intersection points (the green points *p*(*i*)) between the Hénon attractor and the stable manifold segment (vertical dashed line) passing through the fixed point.

Figure 3(a) shows a portion of the Hénon attractor (black densely dotted “lines”) close to the fixed point, marked as a cyan circle. As mentioned, important for our discussion are the six thin lines, labelled 1, 2, 3, 4, 5, 6, lying on the unstable manifold. We compute the angles between these lines and the stable manifold (red dashed lines). One illustrative angle is indicated in green on the left side of the fixed point in Fig. 3(a). From Fig. 3(a), it is easy to observe that angles are larger (smaller) on the left (right) side of the fixed point. This is nicely manifested by the colors in Fig. 2(d), which show that the angles always decrease for increasing values of *x*, illustrating the high-precision and sensibility of our numerical calculations.

The relevant point here is that lines 3 and 4 are in fact the *same line* because they both join at the fold *F*_34_ indicated in Fig. 3(a). Therefore, segments 3 and 4 are not parallel but approach each other more and more as *x* increases towards *F*_34_. In other words, segment 3 is approaching segment 6 while segment 4 is receding from it. The same occurs for 1 and 2 which join 5 at a point located outside the range of Fig. 3(a). This is possible because 5 is in fact a doublet, say 5_1_, 5_2_, only visible under further magnification. The infinite “lines” are segments connected at an infinite number of folds similar to *F*_34_: segments 1, 2, 3 *approach* 6 while segments 4, 5 *move away* from 6. In terms of the angles between the manifolds, this means that segments 1, 2, 3 approaching 6 have *larger* angles than 4, 5 which are getting away from 6, reproducing what is nicely manifest in Fig. 2(d).

In the next magnification considered by Hénon, Fig. 2(e), we have essentially the same structure of segments if we relabel them 4 → 1′, 5_1_ → 2′, 5_2_ → 3′, 6 → 4′, 5′, 6′. However, in sharp contrast to what happened before, now 1′, 2′, 3′ are getting *away* from 6′ when *x* increases, while 4′, 5′ is getting closer to it. In other words, segments 1′, 2′, 3′ have now angles *smaller* compared to the angles of the segments 4′, 5′ [as observed Fig. 2(e)]. This is precisely the opposite of what happened in Fig. 2(d). Next, in Fig. 2(f), the angles of the new line segments 1″, 2″, 3″ are *larger* compared to the angles of 4″, 5″, as in the Fig. 2(d). We conjecture that this scenario will repeat indefinitely. Summarizing, the tiny angle differences between the layers of dots forming the “curves” of the attractor reflect the simple fact that such layers are not parallel.

Comparing angles in Fig. 2(d,f), we observe that the colors of the upper three thin layers represent angles *smaller* than those of the lower layers. This suggests that a more suitable magnification to evidence self-similarity of position and angles would be to use a magnification allowing one to pass directly from Fig. 2(d) to Fig. 2(f). In the magnifications used by Hénon, each window decreases by 10% from the previous one. As may be seen comparing Fig. 2(a–c), under such constant magnifications the separation between the six thin layers increases more and more, i.e. the aspect ratio between the figures changes. Thus, the question now becomes: Are the angles important for the definition of the self-similarity? What is the *correct* sequence of magnifications that keeps self-similarity for both the position of thin layers and the manifold angles?

**Figure 4:**
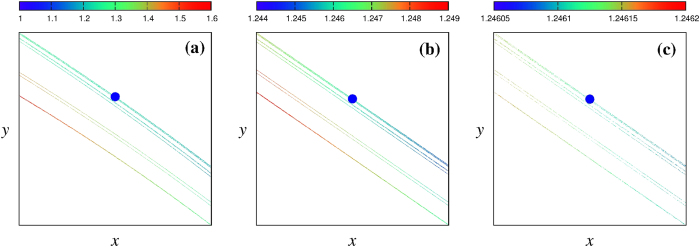
Angle-enhanced magnifications illustrating self-similarity both in the geometry and in the angles of the chaotic attractor close to the fixed point 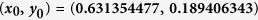. (**a**) magnification of 1.50%, (**b**) 2.10%, (**c**) 2.70%. The windows shown in each panel are defined in Table 1.

To answer these questions we searched for a suitable sequence of magnifications. Since the angle distribution repeats after two Hénon-like magnifications [i.e. when passing from Fig. 2(d) to Fig. 2(f)], a plausible guess is that the magnification should be about one order of magnitude less than 10%. By trial-and-error we found the sequence used in Fig. 4: (a) 1.50%, (b) 2.10% and (c) 2.70%. Table 1 gives the sizes of the three panels in Fig. 4. As expected, the distance between layers is now clearly constant. In addition, in all magnifications the upper three thin layers have larger angles than the lower ones. After successive magnifications one approaches more and more the unstable point (*x*_0_, *y*_0_). The value of *θ*_*num*_ close to this point can be compared with analytical results. The angle between the stable and unstable manifolds may be determined exactly [Methods, Eq. (13)]:


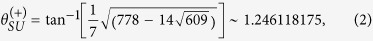


This value lies very close to the center of the interval shown in the colorbar of Fig. 4(c). This nicely shows the good precision of our numerical determination of *θ*_*num*_.

### Measuring how parallel the line segments are

As pointed out by Hénon, close to the fixed point there is an infinity of quasi-parallel line segments. Using the manifold angles it is possible to quantify how parallel these quasi-parallel line segments are. To this end, we locate the intersection points between the line segments 1, 2, 3, 4, 5 and the line segment of the stable manifold which passes exactly at the fixed point. In Fig. 3(b), arrows indicate the first five intersection points *p*(*i*) with *i* = 1, 2, 3, 4, 5 referring to the line segments defined in Fig. 3(a). Our goal is to determine as many intersection points as possible when approaching the fixed point. As seen above, for the second magnification around the fixed point [Fig. 2(b)], we have essentially the same organization of segments as in the first magnification [Fig. 2(a)] if the line segments are relabeled as 4 → 1′, 5_1_ → 2′, 5_2_ → 3′, 6 → 4′, 5′, 6′. The five new intersection points are denoted by *p*(*i*′) (*i*′ = 1′, 2′, 3′, 4′, 5′). In the third magnification, Fig. 2(c), the five new points are denoted by *p*(*i*″), (*i*″ = 1″, 2″, 3″, 4″, 5″), and so on. After *N* + 1 magnifications we have a sequence of 5(*N* + 1) points: 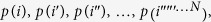  where *N* = 0, 1, 2… gives the numbers of primes which appear in *i*. For simplicity we abbreviate 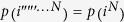. After infinite magnifications, the last intersection point of this sequence will be the fixed point.

For each intersection point of the *p*(*i*) sequence we determine the manifold angles. This allows us to compare the manifold angles between distinct line segments and to measure how parallel they are at the intersection points. For example, let us compare how parallel the pair of line segments 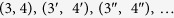 are at the intersection points, when approaching the fixed point. For this purpose we define the manifold angle difference for (*N* + 1) magnifications as


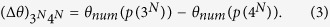


**Figure 5:**
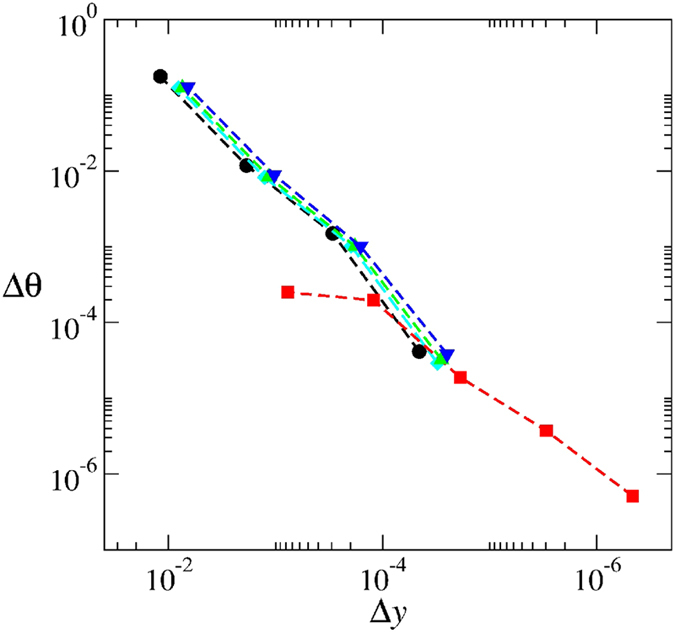
Scaling of the manifold angle difference (Δ*θ*) between distinct line segments at the intersection points as a function of the distance Δ*y* between the intersections points as we approach the fixed point. We use five magnifications and compare the following pairs of intersection points: black circles for 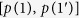, 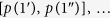, cyan diamonds for 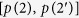, 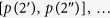, green triangles for 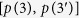, 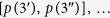, red squares for 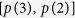, 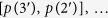, and blue triangles for 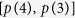, 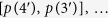. The red curve starts more to the right because points *p*(3) and *p*(2) are closer to each other when compared to the other pairs of points (see Fig. 3).

Here, 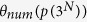 and 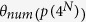 are, respectively, the manifold angles of the intersection points 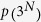 and 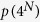 after *N* + 1 magnifications. Table 2 collects the numerical values of the points 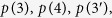

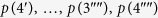
, together with their corresponding manifold angles. Figure 5 shows a log-log plot of 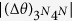 (blue triangle) as a function of 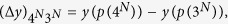  the distance between the intersection points in the *y* variable. The plot clearly shows that the manifold angle difference between the pairs of intersections points [*p*(3), *p*(4)], 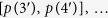 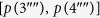, goes to zero as one approaches the fixed point. The manifold angles difference for other pairs of line segments were also compared at the intersection points and are shown in the same figure: 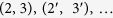 (red squares); 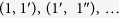 (black circles); 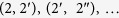 (cyan diamonds); and 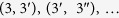 (green triangles). The intersection points and manifold angles for these cases are also presented in Table 2. In all cases considered, one sees that pairs of line segments at the intersection points become more and more parallel as they approach the fixed point. Furthermore, a power-law decay is observed in all cases, but with two different decay rates: a decay 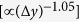 for the pairs 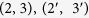, …. For all other pairs the decay rate is 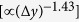. The last column in Table 2 nicely shows that the manifold angles at the intersection points for a line segment *k* (=1, 2, 3) satisfy:


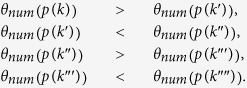


In addition, from Table 2 one sees that comparing the above angles with the manifold angle 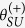 at the fixed point (last line in Table 2), the angles obey


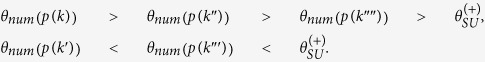


Thus, the manifold angles at the intersection points *alternate* as the magnification increases, being successively *larger* and *smaller* than 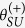. In other words, they oscillate around 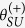 as one moves towards the fixed point. Note that this alternation is the reason why the magnifications from Fig. 2(d–f) had to be replaced by the correct magnification from Fig. 4(a–c). After a large number (*N* → ∞) of magnifications, the manifold angles at the intersection points converge more and more to 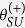 and the line segments should become parallel between themselves and with the unstable direction at the fixed point.

From a technical point of view, we mention that to determine the intersection points we evolve the Hénon map for 2 × 10^11^ iterations and determine when the point (*x*, *y*) of the attractor intersects the line segment of the stable manifold with a precision of ~10^−9^. After this, plotting the intersection points and sucessive magnifications around the fixed point, it is not difficult to associate the intersection points with their corresponding line segments.

### Angle-enhanced bifurcation diagrams

**Figure 6:**
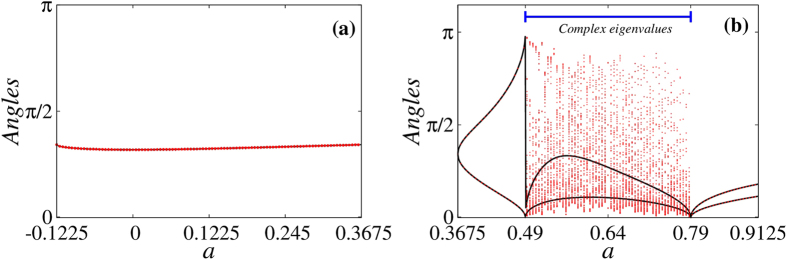
Angle *θ*_*num*_ (red points) as a function of *a* compared with the analytical results for the cases: (a) 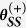 (black line) for period-1. Both curves overlap perfectly. (**b**) 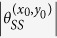 and 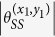 (black lines) for period-2. The curves only do not coincide in the region of complex eigenvalues. Here *b* = 0.3.

Analytical results for period-1 and period-2 (see Methods) allow us to study the behavior of manifold angles along bifurcation diagrams. When decorated by manifold angles, we refer to such diagrams as *angle-enhanced* bifurcation diagrams. Figure 6(a) compares the analytical angle 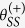 [Methods, Eq. (13)] for period-1 with *θ*_*num*_ as a function of *a* inside the stability domain 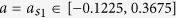 for *b* = 0.3. Both angles overlap identically showing that the numerical procedure can be efficiently used to extract the angles between stable-stable manifolds. It also describes correctly what happens at both ends of the interval, *a* = −0.1225 and *a* = 0.3675, where bifurcations occur.

In Fig. 6(b) we plot the exact angles 
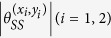
 – see Methods, Eqs (23) and (24) – and *θ*_*num*_, as a function of *a* inside the stability domain 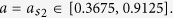 This stability domain has a sub-interval 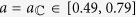 inside which the eigenvalues of the Jacobian matrix are complex and no manifolds exist, so that *all* trajectories around the orbital points change their directions, forming a kind of “rotating dynamics” (see Methods for more details). Figure 6(b) clearly shows that, outside the sub-interval 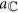, the angle *θ*_*num*_ is in perfect agreement with the analytical result for 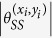. For each value of *a* outside 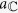, *θ*_*num*_ oscillates periodically in time between 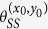 and 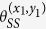. When the parameter *a* approaches 0.49 from below and 0.79 from above, all angles approach 0 or *π*. In other words, the stable manifolds become either parallel or anti-parallel when approaching the limits of the sub-interval with complex eigenvalues. Inside 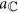 the numerical procedure disagrees with the analytical one, and a distribution of angles *θ*_*num*_ is observed for *each* value of *a*. In fact, what happens is that *θ*_*num*_ varies non-periodically in time and does not converge to a final value, since there is no invariant manifold. This is a consequence of the aforementioned rotating dynamics around stable orbital points, with the numerical procedure generating many angles *θ*_*num*_.

**Figure 7:**
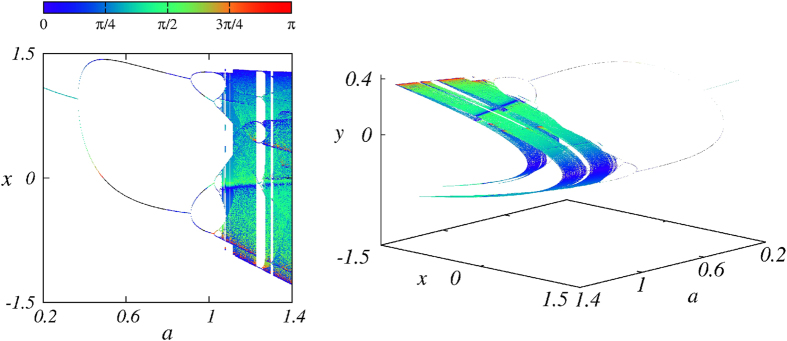
Two representations of angle-enhanced bifurcation diagrams. Left: two-dimensional diagram, and Right: three-dimensional diagram. Black lines are used when complex eigenvalues of the Jacobian matrix are expected. For a video animation of the three-dimensional bifurcation diagram see the Supplementary Material. Here *b* = 0.3.

Figure 7 shows explicit examples of two- and three-dimensional angle-enhanced bifurcation diagrams for *b* = 0.3 and 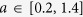. The colorbar indicates the colors assigned to the angles of every point. Rotating dynamics is plotted using black dots. In Fig. 7 one recognizes readily the specific locations characterized by the most interesting angles 0 and *π*. The leftmost panel in Fig. 7 shows the location of the tangencies (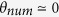 or 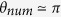) at the outer borders of the diagram at several specific inner positions. These positions correspond to folds in the three-dimensional representation of the bifurcation diagram seen on the rightmost panel in Fig. 7. Much more detailed views of the inner structure of the attractor and the recurrent regions of color densification associated with the distribution of angles can be seen in a video included as Supplementary Material. This video shows how the different sheets of the foliation composing the attractor are folded and superposed in a complicated way.

The comparison between numerical and analytical results shows that it is possible to determine manifold angles *θ*_*num*_ for *stable* orbits at bifurcating points and use them to detect a rotating dynamics (i.e. complex eigenvalues). A very interesting application of this possibility is to use it to record angles between manifolds when computing bifurcation diagrams. By dressing each point of such diagrams with a color corresponding to an angle one can transform the usual uniformly gray bifurcation diagrams into a colorful representation providing not only the same familiar informations as before, but also direct visual access to big-data informations, namely manifold angles at every point.

## Discussion

This work explored practical informations that can be extracted from manifold angles over extended parameter intervals. The angles between stable-stable and stable-unstable manifolds of the Hénon map were determined analytically and numerically. We showed how the use of manifold angles can be turned into a useful tool not to prove but to represent more accurately the apparent self-similarity of chaotic attractors. This was demonstrated explicitly by quantifying the degree of parallelism of the “curves” forming the original Hénon attractor, and allowed us to discover the appropriate magnification sequence needed to display properly the self-similarity of the attractor. While in the original work of Hénon the quasi-parallel curves change their phase-space inclination after every magnification, our manifold-angle analysis allows us to obtain magnifications that consistently preserve the inclination of the quasi-parallel curves. In addition, we derived exact analytical results for period-1 and period-2 angles and compared them with the similar angles extracted numerically. Results are in perfect agreement as long as the eigenvalues of the Jacobian matrix are real. For complex eigenvalues of periodic motions we found manifold angles to oscillate non-periodically in time and to display a peculiar rotating dynamics around orbital points. We pointed out that, self-similarity cannot exist in a strict sense as long as manifold segments are not *de facto* parallel. Examples of angle-enhanced bifurcation diagrams were given both in figures and in a video, illustrating that they convey much more information then conventional diagrams, without angle information. Enhanced diagrams allow direct visualization of the complex geometrical structure of attractors. Such diagrams may be also used for arbitrary dynamical systems. In this context, an interesting open question now is to find out if the angle distribution is *structurally stable*, namely whether or not the distributions in Fig. 7 and in the video remain invariant under *C*^1^-small perturbations and, if not, to try to classify the angle distributions in existence.

## Methods

A great advantage of using the Hénon map is that it allows obtaining exact analytical expressions for manifold angles of trajectories of low periods. Our goal here is to determine such angles analytically for orbits of period 1 and 2 as a function of control parameters. Of significance is to have the possibility of comparing *exact results* with numerical computations of *θ*_*num*_. In the literature, Lyapunov vectors are routinely applied only to chaotic attractors, objects not possible to address analytically.

An added bonus is that our exact formulas allow one to learn what happens with angles between stable-stable (SS) and stable-unstable (SU) manifolds across bifurcations by following the evolution of orbital stability continuously over wide parameter intervals. Thus far, it seems that only the angles between stable and unstable manifolds have been considered in the literature (see however, refs 32,33). Our exact formulas throw light on an unexplored object: intersection angles between *pairs of stable manifolds*.

**Period-1.** The coordinates of the two fixed points 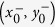 and 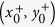 are


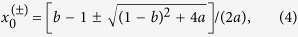



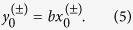


Introducing the abbreviations 
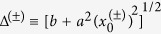
, the eigenvalues of the associated Jacobian matrix may be written as


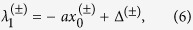



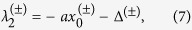


the corresponding eigenvectors being 
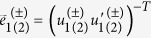
. For *λ*_1_ the components are


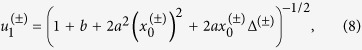



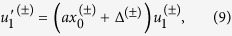


while for *λ*_2_ they are


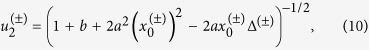



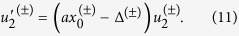


From this, we obtain the angles of the eigenvectors relative to the *x*-axis:


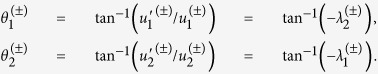


Finally, the angle between manifolds is obtained from


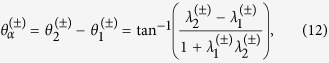


where *α* = *SS*, *SU* denotes, respectively, the stable-stable and stable-unstable manifolds, whose existence depends on the particular choice of the parameters (*a*, *b*). For example, for parameters for which the point 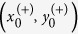 is stable, we use *α* = *SS* due to the presence of two stable manifolds, denoting by 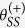 the angle between them. In terms of the control parameters we find


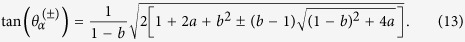


To analyze the behavior of 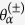 as a function (*a*, *b*), it is necessary to define the boundaries for the domains of stability of 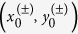. Such boundaries are^34^,^35^:


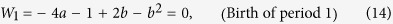



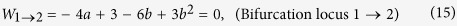


obtained by solving 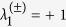 and 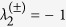, respectively. The stability domain, obtained from Eqs (14) and (15), lies in the interval 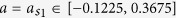 where 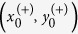 is stable. No complex eigenvalues exist inside this interval.

**Period-2.** The period-2 points (*x*_0_, *y*_0_) → (*x*_1_, *y*_1_) → (*x*_0_, *y*_0_) are located at


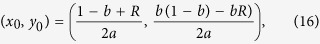



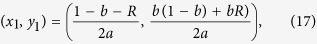


where 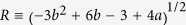. The eigenvalues of the twice-iterated Jacobian matrix are


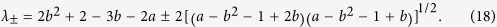


The corresponding eigenvectors are denoted by 
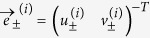
, where *i* = 0, 1 refers to (*x*_0_, *y*_0_) and (*x*_1_, *y*_1_), respectively. For (*x*_0_, *y*_0_) the components are


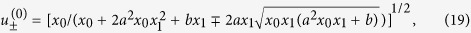



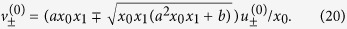


In a similar way, for (*x*_1_, *y*_1_) one has


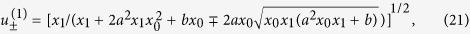



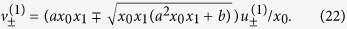


From this we obtain the angles of the eigenvectors relative to the *x*-axis: 
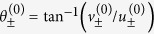
 and 
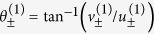
. Finally the angles between manifolds can be written as


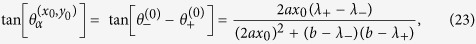



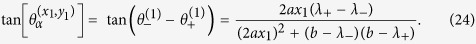


Therefore, the manifolds of each orbital point will have distinct directions as long as *x*_0_ ≠ *x*_1_. As in Eq. (12) for period-1, Eqs (23) and (24) show that the manifolds become parallel (or anti-parallel) when the eigenvalues coincide. The stability boundaries for period-2 orbits are^34^,^35^


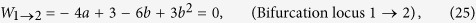



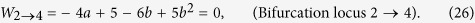


While both orbital points remain stable inside the domain delimited by these equations, the eigenvalues change with parameters and can become complex. To see this in more details we consider once again the classical value *b* = 0.3. Solving Eqs (25) and (26) we obtain the interval 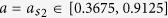. Eigenvalues become complex inside the sub-interval 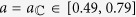, obtained from Eq. (18). Inside the interval 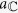 the eigenvalues can be written as


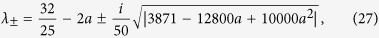


so that their real parts are equal, *i.e.* 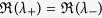. Accordingly, the angle between the stable manifolds is also complex and satisfies 
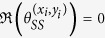
 and 
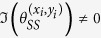
 (*i* = 0, 1). Such complex angles must be correctly interpreted. For this we recall that when eigenvalues and eigenvectors are real, the eigenvectors give the directions along which trajectories near the stable point do not change direction. Such directions are the invariant manifolds, stable or unstable. On the other hand, when the eigenvalues are complex, the eigenvectors are also complex and there are no directions in which the nearby trajectories do not change direction. In fact, such trajectories stay in an invariant plane defined by 
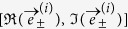
, and 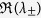 is related to a growth or decay factor, while 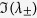 is the strength of the angular rotation velocity on the invariant plane. For 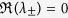, Eq. (27) gives *a* = 0.64, and the dynamics in this plane is an ellipse. Loosely speaking, in the complex case *all* trajectories around the orbital points change directions, forming a kind of “rotating dynamics” and it is not possible to define a manifold. This lack of convergence may be conveniently used to detect regions of rotating dynamics in generic dissipative systems. We mention that non-periodic oscillations of *θ*_*num*_ for a fixed value of *a* inside 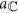 occur for a stable orbit of period 2. This should not be confused with what happens in the chaotic motion, where *θ*_*num*_ also oscillates non-periodically, but each value of the angle is related to one point on the chaotic attractor. Summarizing, if *non-periodic* oscillations of *θ*_*num*_ are observed when the dynamics is periodic, then one is facing rotating dynamics, *i.e.* the presence of complex eigenvalues. We observed that the region of rotating dynamics contains also narrow windows, e.g. near *a* = 0.64, where *θ*_*num*_ oscillates periodically, but with a period twice the orbital period. The origin of such entrainments is not yet understood.

## Additional Information

**How to cite this article**: Beims, M. W. and Gallas, J. A. C. Manifold angles, the concept of self-similarity, and angle-enhanced bifurcation diagrams. *Sci. Rep.* **6**, 18859; doi: 10.1038/srep18859 (2016).

## Supplementary Material

Supplementary Information

Supplementary video

## Figures and Tables

**Table 1:** Windows of the magnifications of Hénon attractor shown in Fig. 4 (see Text).

Magnification	(*x*_*min*_, *x*_*max*_)	(*y*_*min*_, *y*_*max*_)
*a* (1.50%)	(0.612172196, 0.650536758)	(0.183651659, 0.195161027)
*b* (2.10%)	(0.630951649, 0.631757305)	(0.189285495, 0.189527191)
*c* (2.70%)	(0.631343601, 0.631365353)	(0.189403080, 0.189409606)

**Table 2:** Manifold angles at the intersection points between the unstable attractor and one line segment of the stable manifold which passes exactly through the fixed point.

Intersection points	[*x*(*p*(*i*)), *y*(*p*(*i*))]	*θ*_*num*_(*p*(*i*))
*p*(1)	[0.623991679, 0.175242305]	1.413345871
*p*(2)	[0.626378625, 0.179834157]	1.364126599
*p*(3)	[0.626778459, 0.180603340]	1.364376797
*p*(4) = *p*(1′)	[0.630199980, 0.187185451]	1.235756109
*p*(5_1_) = *p*(2′)	[0.630575866, 0.187908554]	1.238890872
*p*(5_2_) = *p*(3′)	[0.630639210, 0.188030422]	1.238694445
*p*(4′) = *p*(1″)	[0.631174268, 0.189059734]	1.247590954
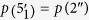	[0.631232967, 0.189172650]	1.247125711
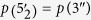	[0.631242762, 0.189191498]	1.247106895
*p*(4″) = *p*(1″′)	[0.631326362, 0.189352312]	1.246093647
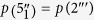	[0.631335505, 0.189369990]	1.246100471
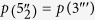	[0.631337044, 0.189372867]	1.246096737
*p*(4″′) = *p*(1″″)	[0.631350066, 0.189397917]	1.246134991
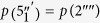	[0.631351493, 0.189400663]	1.246129610
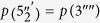	[0.631351736, 0.189401124]	1.246129101
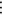	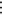	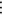
Fixed point (*p*(6^∞^))	[0.631354477, 0.189406343]	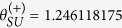

At each magnification we have a proliferation of intersection points so that at the first magnification points are relabeled as *p*(4) → *p*(1′), *p*(5_1_) → *p*(2′), *p*(5_2_) → *p*(3′), *p*(6) → *p*(4′), *p*(5′), *p*(6′); for the second magnification relabeled as 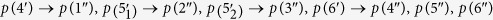, and so on. Note that we do not worry about the points 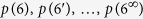, since after magnifications they proliferate and we just extract the new points 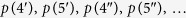 from them. In fact, the last point *p*(6^∞^) after infinite magnification is the fixed point.
