# Supplementary material for: Manifold angles, the concept of self-similarity, and angle-enhanced bifurcation diagrams
Source: Sci Rep. 2016 Jan 6;6:18859. doi: 10.1038/srep18859 (PMC4702165; doi:10.1038/srep18859)
Supplement: Supplementary Information [file srep18859-s1.pdf]

Manifold angles, the concept of self-similarity, and angle-enhanced bifurcation diagrams – Video legend

Marcus W. Beims[1,2,3,4] **and Jason A.C. Gallas**[2,3,4,5,*]

[1]Departamento de Física, Universidade Federal do Paraná, 81531-990 Curitiba, Brazil
[2]Departamento de Física, Universidade Federal da Paraíba, 58051-970 João Pessoa, Brazil
[3]Instituto de Altos Estudos da Paraíba, Rua Infante Dom Henrique 100-1801, 58039-150 João Pessoa, Brazil
[4]Max Planck Institute for the Physics of Complex Systems, Nöthnitzer Str. 38, 01187 Dresden, Germany
[5]Institute for Multiscale Simulation, Friedrich-Alexander-Universität Erlangen-Nürnberg, 91052 Erlangen, Germany
[*]jason.gallas@cbi.uni-erlangen.de

ABSTRACT

This Supplementary Information provides the video legend

Video legend

The video shows an example of a three-dimensional angle-enhanced bifurcation diagrams for $b = 0.3$ and $a \in [0.2, 1.4]$. The color coding used is described on the left panel in Fig. 7 of the main text. Rotating dynamics is plotted with black dots. From this video one recognizes readily the specific locations characterized by the most interesting angles 0 and π. These positions correspond to folds in the three-dimensional representation of the bifurcation diagram seen on the rightmost panel in Fig. 7 of the main text. The video presents detailed views of the inner structure of the attractor and the recurrent regions of color densification associated with the distribution of angles. This video shows how the different sheets of the foliation composing the attractor are folded and superposed in a complicated way.
