# Supplementary figures and images for: Manifold angles, the concept of self-similarity, and angle-enhanced bifurcation diagrams
Source: Sci Rep. 2016 Jan 6;6:18859. doi: 10.1038/srep18859 (PMC4702165; doi:10.1038/srep18859)

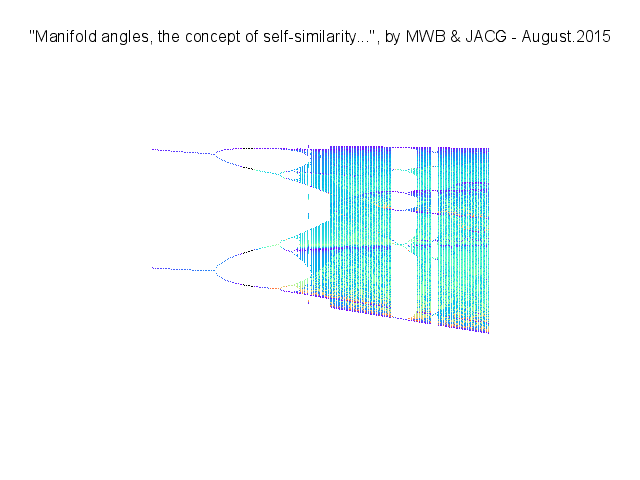

Supplement: Supplementary video [file srep18859-s2.gif]
